# Effects of Brash Removal After Clear Felling on Soil and Soil-Solution Chemistry and Field-Layer Biomass in an Experimental Nitrogen Gradient

**DOI:** 10.1100/tsw.2001.93

**Published:** 2001-10-12

**Authors:** E. Ring, L. Hogbom, H.A. Nohrstedt

**Affiliations:** SkogForsk-The Forestry Research Institute of Sweden, Uppsala Science Park, Uppsala, Sweden

**Keywords:** chemistry, clearcut, forest fertilisation, field layer, nitrogen, soil solution, soil

## Abstract

Biofuels, such as brash from forest fellings, have been proposed as an alternative energy source. Brash removal may affect the sustainability of forest production, e.g., through a change in the availability of cations and N in the soil. We report initial effects of brash removal on inorganic N content in humus and mineral soil, soil-solution chemistry, and field-layer biomass after clear felling an N-fertilisation experiment in central Sweden. The experiment comprised six different fertiliser levels, ranging from 0 to 600 kg N ha(-1). Urea was given every 5th year during 1967 to 1982 to replicated plots, giving total doses of 0 to 2400 kg N ha(-1). Clear felling took place in 1995, 13 years after the last fertilisation. The removal of brash decreased the NO3- content in the humus layer after clear felling. A decrease in the NO3- concentration of the soil solution was indicated during most of the study period as well. No effect of the previous N fertilisation was found in the humus layer, but in the mineral soil there was an increase in NO3- content for the highest N dose after clear felling ( p = 0.06). The soil-solution chemistry and the field-layer biomass showed an irregular pattern with no consistent effects of brash removal or previous fertilisation.

### INTRODUCTION

Biofuels, such as brash from forest fellings, have been proposed as an alternative to fossil fuels and nuclear energy. Whole-tree harvesting (WTH), i.e., harvesting of all aboveground parts, is common in southern Sweden[1]. However, WTH, which includes harvesting of brash (tops, branches, and needles), might affect the long-term sustainability of forest production and cause unwanted environmental effects. Several studies indicate that WTH poses a greater threat to the long-term sustainability of forest production than conventional stem harvesting, which leaves brash on site, because of an increased nutrient removal[2,3]. A 1.5 to 5 times increase in the nutrient removal has been estimated after WTH compared with more conventional stem harvesting[4]. Despite the large increase in nutrient removal, this makes up only a fraction of the total nutrient supply of a site.

Clear felling of a forest is a considerable disturbance that affects the nutrient cycling, the runoff, and the microclimate of a site. Increases in the $NO_3^-$ availability in the soil[5] and in the field-layer plant biomass[6] typically follow clear felling. $NO_3^-$ production exceeding the uptake by plants and soil organisms is likely to be lost by leaching[7,8].

Nitrification in acid forest soils is likely to be controlled by substrate availability, pH, water potential, nutrient status, and soil temperature[5,8]. Each of these factors is affected by clear felling and whether the brash is left on site or removed. For instance, on a clearcut with sparse field-layer vegetation, brash removal increased the temperature and decreased the water content of the humus layer during the growing season following clear felling[9]. Some studies on brash removal after clear felling have shown a decrease in $NO_3^-$ leaching or concentration after brash removal compared with when the brash was left on site[10,11]. The lower leaching of $NO_3^-$ from brash-free areas compared with brash-covered areas might have been caused by a less favourable microclimate for mineralisation and nitrification in the absence of brash[10]. The field-layer vegetation has been reported to increase after brash removal, which might influence the N retention as well[10,12].

Forest fertilisation with N increases the storage of N and organic matter (OM) in boreal ecosystems[13,14,15]. Because of the greater buildup of N compared with C in relative terms, the C to N ratio of the soil's OM decreases[13,14,15]. N mineral-isation and nitrification in forest soils are related to the C to N ratio[16]. Previous fertilisations with N may further increase $NO_3^-$ leaching after clear felling[17,18], partly because of a lowering of the C to N ratio[19].

In the late 1960s, the forest fertilisation experiment D11 Mangskog was started in central Sweden in order to study effects of urea fertilisation on tree-stem growth. Four applications of 0 to 600 kg urea-N $ha^{-1}$ were made at 5-year intervals, giving total N doses ranging from 0 to 2400 kg $ha^{-1}$. The present study was initiated 13 years after the last fertilisation when the Mangskog experiment was to be clear-felled. Our aim was to determine the effects of clear felling of previously N fertilised forests with and without brash removal on the N dynamics of the soil and the development of the field-layer biomass.

### MATERIAL AND METHODS

#### Site and Experimental Description

The D11 Mangskog experiment is located on a 10% west-facing slope in western-central Sweden (59°50′N/12°20′E, 175 m a.s.l.)[20,21]. The annual mean air temperature in the area is 5.3°C and the annual precipitation 640 mm. The soil is podzolised and it has developed on a sandy–stoney–gravelly till. The thickness of the humus layer is 4 to 5 cm. The site is rather fertile, having a site-quality class of 9.3 $m^3$ $ha^{-1}$ $year^{-1}$ (control plots). The dominant tree species was Norway spruce *(Picea abies)*. The experiment was established in 1967 to examine the effects on tree-stem growth of repeated fertilisations with urea-N. Between 1967 and 1982, urea-N was applied four times, using six dosages (0, 120, 240, 360, 480, and 600 kg N $ha^{-1}$) at 5-year intervals. Thus, the plots were fertilised at totals of 0, 480, 960, 1440, 1920, and 2400 kg N $ha^{-1}$. Each fertiliser treatment was replicated twice within a randomised block design. Hereafter, the fertiliser treatments are referred to with reference to the total fertiliser dose, i.e., 0N-2400N.

Stem-wood growth was not significantly affected by the fertilisations, and no residual changes of the nutrient concentrations in the aboveground parts of the trees were observed 13 years after the last fertilisation[20]. However, residual effects were observed in the soil[21] and in the cover of some field- and bottom-layer species[22]. In the humus layer, the concentration of N increased and the C to N ratio decreased with increasing fertiliser dose (from about 25 to 20)[21]. The calculated recovery of added N was 100% for the lowest fertiliser dose and 25 to 50% for higher doses. The recovery of N in the different treatments was the amount of N in the stand, the field layer, the organic layer, and in the upper 10 cm of the mineral soil less the corresponding amount in one control plot.

Before clear felling, each main plot (30 × 35 $m^2$) was divided into two subplots (30 × 17.5 $m^2$) on which the brash was to be either left or removed (hereafter referred to as the brash treatment). The amount of brash left on the different subplots varied between 57 and 85 Mg $ha^{-1}$, and the corresponding variation in the storage of elements in the brash was (min-max values in kg element $ha^{-1}$): 360 to 530 N, 30 to 50 P, 28 to 43 S, 220 to 510 Ca, 34 to 45 Mg, 160 to 220 K, and 45 to 71 Mn (S. Jacobson, SkogForsk, personal communication, 2001). The experimental site was clear felled in the autumn of 1995. Without entering the plots, a logging machine harvested the exterior parts of the plots, whereas the interior parts were manually felled. On the subplots that were to remain covered by brash, the brash was left on site and evenly distributed over the subplot from which it came. For the subplots without brash, the trees were processed outside the plots. This resulted in a split-plot design with fertiliser dose as the main plot treatment and brash treatment as the subplot treatment. The Mangskog site is further described in references[20,21]

#### Soil Sampling

Soil samples for estimating the inorganic N pools were taken by coring in September 1997, 1998, and 1999. The humus layer was sampled with a soil auger (Ø = 61 mm in 1997 and 1998, and Ø = 70 mm in 1999), and the mineral soil (0 to 10 cm) was sampled with a corer (Ø = 27 mm); 12 samples per subplot were taken from the humus layer and mineral soil, respectively. Treatment 2400N in block B was omitted in all 3 years because of high groundwater levels. In 1997, the treatments 960N and 1440N in block B were omitted as well. The soil material was sieved (mesh size 5.6 mm for humus samples and 4 mm for mineral soil samples) and pooled subplot-wise in order to create composite samples representing each subplot. The soil samples were kept frozen until they were chemically analysed at the Department of Soil Sciences, Swedish University of Agricultural Sciences, Uppsala. The freezing of samples probably released some N. This release was regarded as a systematic error. The concentrations of $NO_3^-$ and $NH_4^+$ were analysed colorimetrically by means of an AutoAnalyzer TRAACS 800 after extraction by 1 *M* KCl. The ratio between soil and salt solution was 1:2.5 by volume. In a soil study performed in 1995, treatment 0N in block A was omitted because the plot was considered to deviate significantly from the other plots already before clear felling[21]. In the present study, both 0N plots were used since the variation within the 0N treatment was of the same order as the variation within the other treatments.

#### Soil-Solution Sampling

Soil solution was sampled by means of ceramic suction cups (type P80) at 30- to 40-cm depth in the mineral soil. In October 1994, three cups per subplot were centrally installed about 7 m apart on a line parallel to one side of the subplot. The cups were installed in the soil at an angle of approximately 60°, leaving the soil right above the cup undisturbed. Each cup was attached by a plastic tube to a PVC container ($Ø_{outer}$ = 50 mm, length = 30 cm), which was largely buried in the soil less than 1 m away from the cup. A few days before sampling, a suction of 70 to 80 kPa was applied by hand pumping. At sampling, equal amounts of soil solution extracted by each of the three cups within each subplot were mixed in order to create a composite sample representing the subplot. The soil-solution samples were frozen as soon as possible after sampling.

The soil solution was analysed for electrical conductivity, pH, $Ca^{2+}$, $Mg^{2+}$, $K^+$, total Mn, total Al, inorganic N, total N, and $SO_4^{2-}$ by AL control AB in Uppsala. Organic N was calculated as the difference between total N and inorganic N. Occasionally, this subtraction gave negative values for organic N, which were set at zero. Concentrations less the chemical detection limit (mainly affecting $NH_4^+$ and $NO_3^-$) were set equal to the detection limit. For the inorganic N species, the detection limit varied between 1 and 5 µg N $l^{-1}$.

Soil solution was collected three to five times during the growing seasons of 1995 to 2000. Before clear felling in 1995, 5 samplings were performed and 22 samplings were performed after clear felling. The soil-solution sampling was somewhat problematic at the Mangskog experiment. The main reasons were the great stoniness of the soil and an occasionally high groundwater table, both of which complicated the installation of the suction cups. Suction cups were therefore only installed in treatments 0N (blocks A and B), 480N (block A), 960N (blocks A and B), 1440N (block A), 1920N (blocks A and B), and 2400N (block A). Regarding the variation between the two 0N plots acknowledged in a previous soil study[21], considerable deviations in the soil-solution concentration between the 0N plots were found for $Ca^{2+}$, $Mg^{2+}$, and $K^+$ in the subplots with brash left on site and for pH in the subplots with brash removed. About 40% of the samples were slightly contaminated by OM, sand, or undefined white flakes. Nevertheless, these observations were included in the statistical analyses. Moreover, some cups failed to extract water resulting in incomplete composite samples from some subplots. Thus, of the total number of samples, about 56% were complete composite samples, 3% were made up by water extracted from only one sampler, 39% were made up by water extracted from only two samplers, and 2% were missing or excluded because of small sample volumes.

At soil-solution sampling, the groundwater table was monitored in one tube per subplot from 1997 and onwards. The groundwater tube was installed adjacent to the suction cup, which was most prone to be affected by a high groundwater table. Our measurements revealed that the water samples from the following subplots probably contained some groundwater (mean groundwater-table ± standard deviation): 0N in both brash treatments in block A (brash removed: 30 ± 7 cm, brash left: 43 ± 9 cm), and in brash removed in block B (45 ± 9 cm), 960N in brash left in block B (35 ± 16 cm), 1920N (46 ± 6 cm) in brash left in block A, and 2400N (25 ± 15 cm) in brash removed in block A.

### Field-Layer Biomass

The field-layer biomass was destructively sampled every consecutive September between 1997 and 2000. The same plots were omitted for sampling of the field-layer biomass as were omitted for sampling of the soil. The sampling in 2000 followed the same pattern as in 1998 and 1999. On each subplot, six samples of the field-layer vegetation were taken systematically, each sample covering 0.25 $m^2$. No separation into species was made. Common species on the plots after clear felling were *Calamagrostis arundinacea*, *Deschampsia flexuosa, Galeopsis tetrahit, Rubus idaeus,* and *Senecio sylvaticus*. (Nomenclature for vascular plants follows[23].)

### Statistical Analysis

The effects on soil, soil-solution chemistry, and field-layer biomass were analysed by a mixed linear model[24] including these fixed factors: brash treatment; fertiliser dose; interaction between brash treatment and fertiliser dose; sampling date; interaction between sampling date and brash treatment; interaction between sampling date and fertiliser dose; and interaction between brash treatment, fertiliser dose, and sampling date; and the random factors block and interaction between block and brash treatment within fertiliser dose. In order to account for repeated measurements a first order autoregressive covariance structure on residuals was used. The denominator degrees of freedom was calculated according to the containment method[24], which in most cases yielded similar results as a general Satterthwaite approximation[24]. As regards to the soil-solution chemistry, this model was applied both for the pre- (1995) and postfelling data sets (1996 to 2000). In the analysis of the postfelling soil-solution data, the prefelling mean concentration on each subplot was introduced as a covariable when its $p$ value was ≤ 0.15 in order to include the potential variation between subplots present before clear felling and brash treatment. Thus, a covariable was introduced for pH ($p$ = 0.0014), $NO_3^-$ ($p$ = 0.12) and organic N ($p$ = 0.011). The normality of the distributions of residuals improved after a logarithmic transformation was performed for all chemical variables of the soil solution apart from pH and for $NO_3^-$ and $NH_4^+$ in the humus layer.

## RESULTS

### Soil Inorganic N

In general, both the $NH_4^+$ and $NO_3^-$ amounts in the soil were highest in the 1st year of sampling (1997), but decreased during subsequent years. Neither brash treatment nor fertiliser dose affected the $NH_4^+$ amount in the humus layer or the mineral soil ($p > 0.6$) (Figs. 1 and 2). The brash removal lowered the extractable amounts of $NO_3^-$ in the humus layer ($p$ = 0.009) (Fig. 3). The amount of $NO_3^-$ in the mineral soil was affected by the fertiliser dose ($p$ = 0.038). Comparisons of least-square means, adjusted according to Tukey-Kramer[24], revealed that the amount of $NO_3^-$ in the mineral soil tended to be higher in the 2400N treatment compared with the control ($p$ = 0.06). The effect varied over time ($p$ = 0.002) (Fig. 4).

### Soil-Solution Chemistry

According to the mixed linear model, no residual effects of previous N fertilisations on the measured soil-solution variables were present in the forest in 1995. The effect of fertilisation on organic N changed over time ($p < 0.001$). None of the other N species was significantly affected by previous fertilisations. The measured chemical variables in the soil solution of the subplots were not significantly different before clear felling and brash treatment. This was important for the analyses of the postfelling data.

After clear felling, there was no significant effect of the brash treatment. The data suggested that the removal of the brash

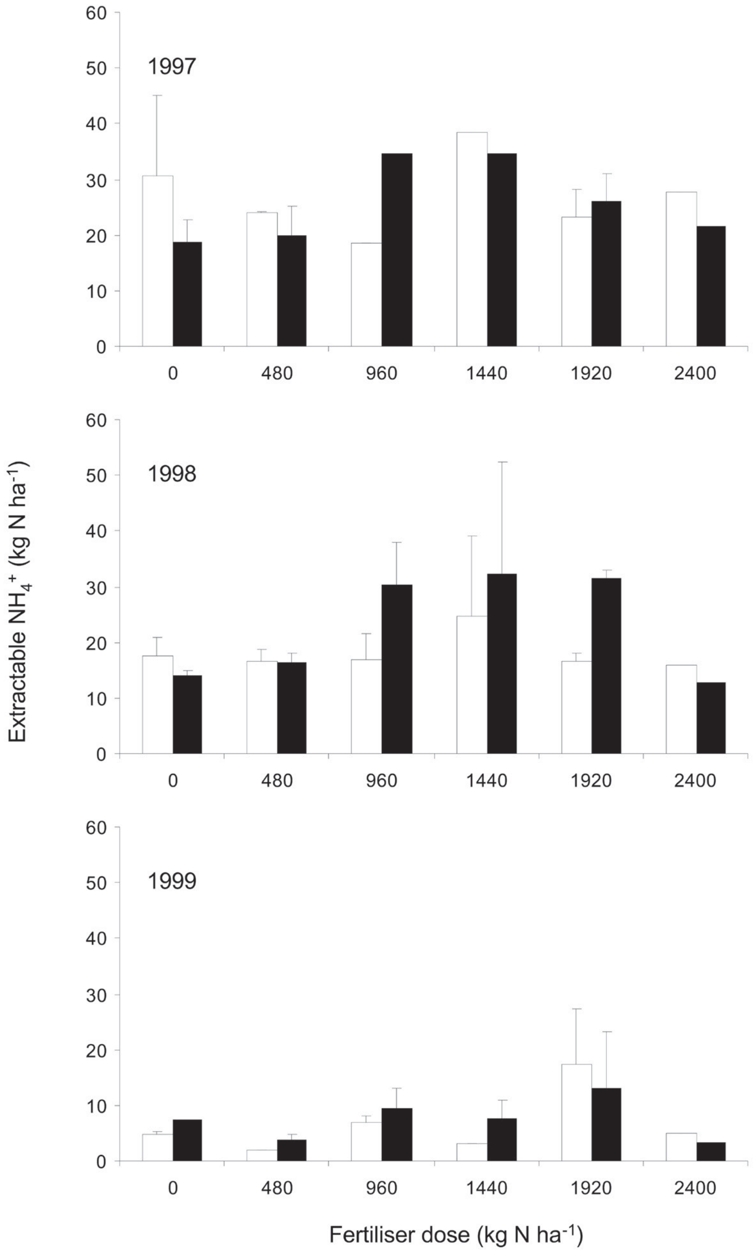

**FIGURE 1.** Extractable amounts of $NH_4^+$-N in the humus layer in 1997, 1998, and 1999. Open bars represent plots with brash removed while closed bars represent plots with brash left on site. Vertical bars indicate +1 s.e. In 1997, the treatments 960N, 1440N, and 2400N are represented by only one block. The 2400N treatment is represented by only one block in 1998 and 1999 as well.

decreased the $NO_3^-$ concentration in the soil solution ($p$ = 0.13) (Fig. 5). The effect of the brash treatment on pH changed with time ($p$ = 0. 02) (Fig. 5). There was a negative relationship ($p < 0.01$, $R^2$ = 0.63 and 0.79) between pH and $NO_3^-$ in both brash treatments. The effect of the brash treatment on $SO_4^{2-}$ changed with time ($p$ = 0.02). Early in the study period, $SO_4^{2-}$ was lowest in the plots where the brash was left. Later on the highest $SO_4^{2-}$ concentrations were observed in the same treatment. $Mg^{2+}$ showed an interaction between fertiliser dose and brash treatment ($p$ = 0.006). Thus, in 0N, 1440N, and 2400N, $Mg^{2+}$ seemed to be higher with brash left than with brash removed, whereas the opposite pattern was seen in 480N, 960N, and 1920N. Mean soil-solution concentrations from 1997 to 1998 of $Ca^{2+}$, $Mg^{2+}$, $K^+$, Mn, $SO_4^{2-}$, and all the N species found in the

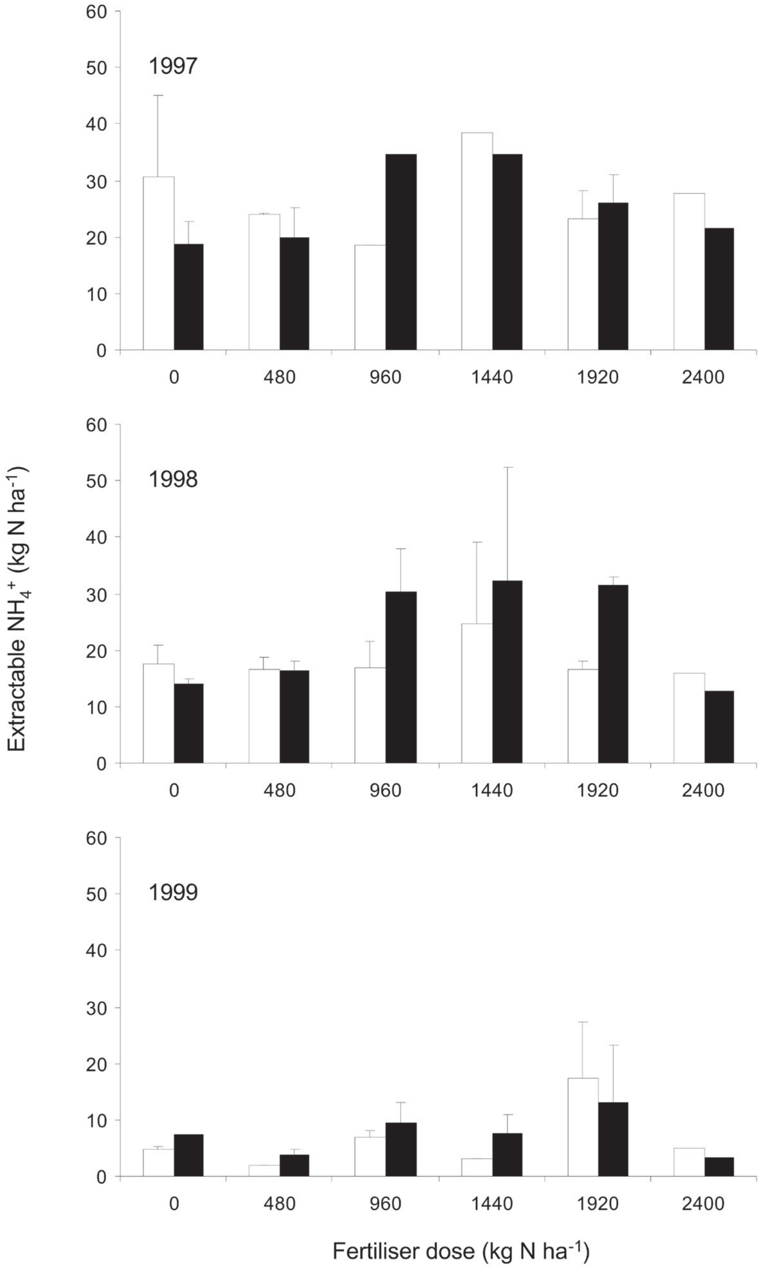

**FIGURE 2.** Extractable amounts of $NH_4^+$-N in the upper 5 cm of the mineral soil in 1997, 1998, and 1999. Open bars represent plots with brash removed while closed bars represent plots with brash left on site. Vertical bars indicate +1 s. e. See also Fig. 1.

plots with brash cover were linearly related to the storage of these elements in the needles at the time of brash treatment. A significant positive relationship was only found between $Ca^{2+}$ in the soil solution and the Ca storage in the needles ($p < 0.05$, $R^2 = 0.53$).

After clear felling, only $Mg^{2+}$ was affected by the previous fertilisations ($p = 0.03$). In 960N, the $Mg^{2+}$ concentration was higher than in the control ($p = 0.05$). The effect of previous fertilisations on $SO_4^{2-}$ changed over time ($p = 0.02$).

In order to test the conformity of the measured $NO_3^-$ concentrations in the soil solution to the $NO_3^-$ storage in the soil, the annual mean $NO_3^-$ concentrations in the soil solution from 1997 to 1999 were linearly related to the observed amounts of $NO_3^-$ in the soil on a subplot level. A weak linear relation-ship with the amount of $NO_3^-$ in the mineral soil was found ($p < 0.05$, $R^2 = 0.11$). Regressing means of the August and September soil-solution samplings with the soil data (taken in September) yielded similar results.

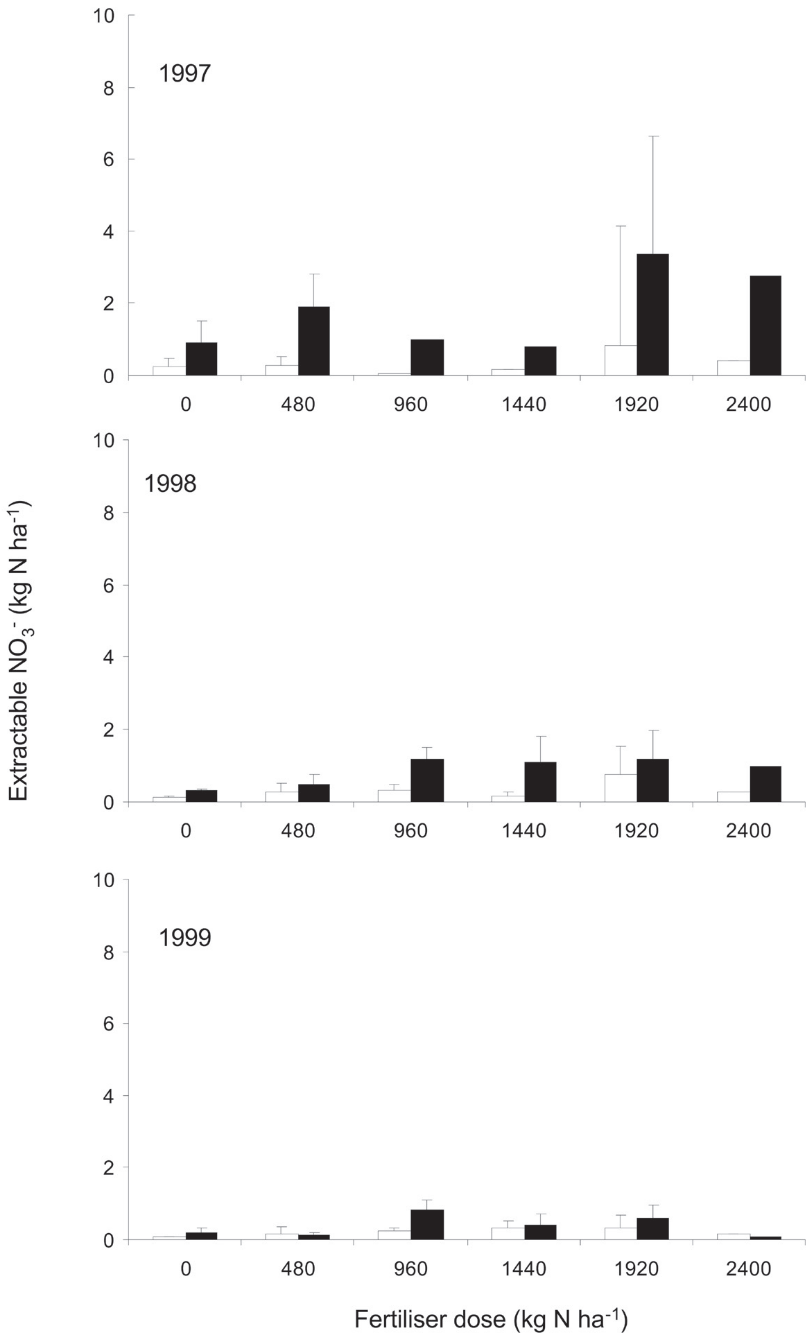

**FIGURE 3.** Extractable amounts of $NO_3^-$-N in the humus layer in 1997, 1998, and 1999. Open bars represent plots with brash removed while closed bars represent plots with brash left on site. Vertical bars indicate +1 s. e. See also Fig. 1.

### Field-Layer Biomass

The field-layer biomass increased rapidly after clear felling (Fig. 6). Neither brash treatment ($p$ = 0.51) nor fertiliser dose ($p$ = 0.78) affected the field-layer biomass.

### DISCUSSION

The brash treatment at the Mangskog experiment affected few of the measured variables. Our results showed a decrease in the amount of $NO_3^-$ in the humus layer following brash removal as

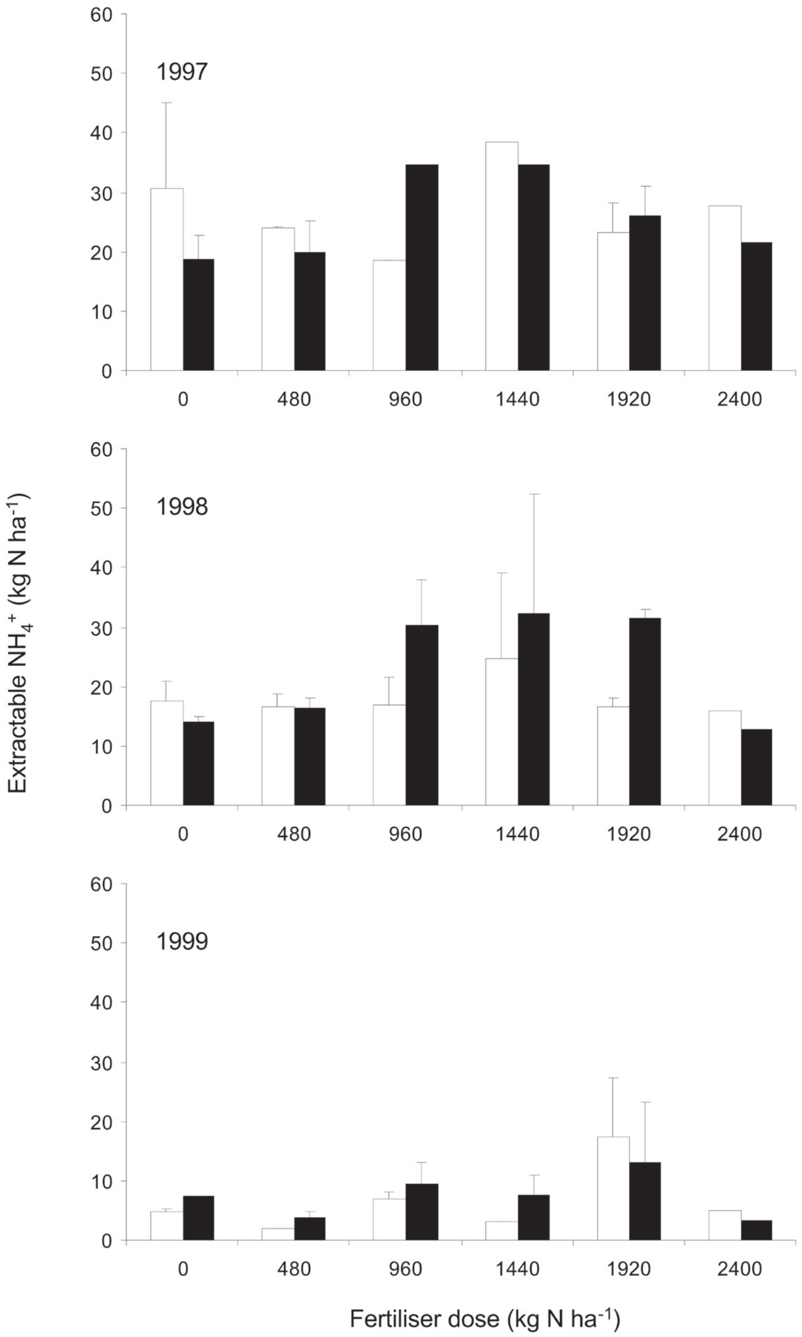

**FIGURE 4.** Extractable amounts of $NO_3^-$-N in the upper 5 cm of the mineral soil in 1997, 1998, and 1999. Open bars represent plots with brash removed while closed bars represent plots with brash left on site. Vertical bars indicate +1 s. e. See also Fig. 1.

compared with brash left on site. This result was supported by a corresponding indicated decrease in the $NO_3^-$ concentration of the soil solution. Other studies have also demonstrated that brash removal might decrease $NO_3^-$ availability[10,11]. The most likely explanation for this decrease in soil $NO_3^-$ availability is that the brash left on site affected the microclimate in favour of the $NH^{4+}$ oxidisers[10]. An alternative hypothesis, that outwash of cations from the fresh litter stimulates nitrification, can probably be ruled out by the fact that the same $NO_3^-$ increase was found under covers of brash and artificial brash (polystyrene beads)[10].

Forest fertilisation with N generally increases the biomass and N storage of the tree crown for some years. At an N-deficient site, the biomass and N storage of branches and needles increased with increasing fertiliser doses[25]. Provided that the amount of

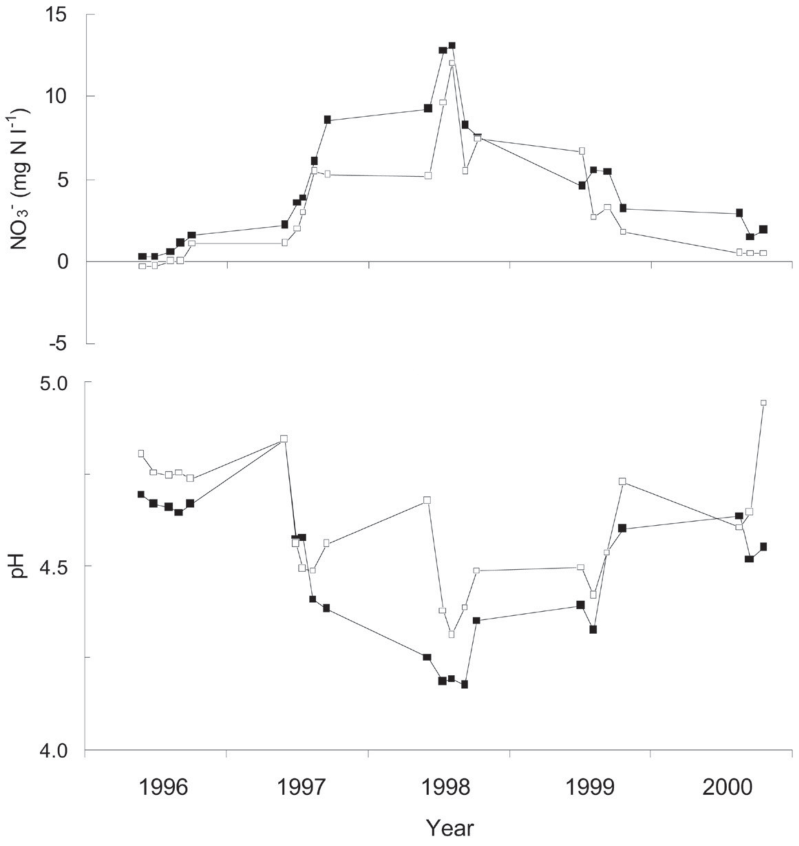

**FIGURE 5.** Least-squares means of the $NO_3^-$-N concentration and pH of the soil solution in the brash treatments after clear felling. Open symbols represent plots with brash removed while closed symbols represent plots with brash left on site.

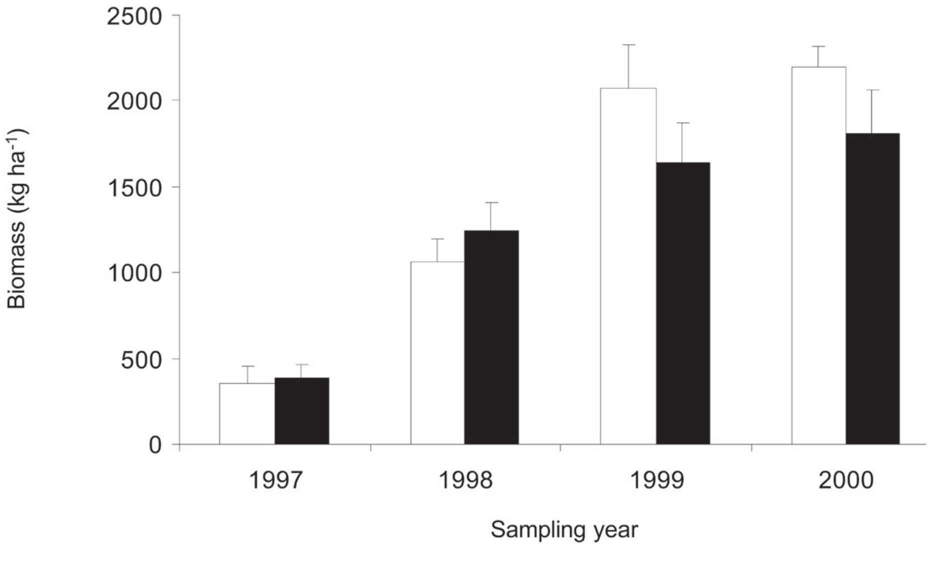

**FIGURE 6.** Development of the field-layer biomass between 1997 and 2000 (means over all fertiliser doses). Open bars represent plots with brash removed while closed bars represent plots with brash left on site. Bars larger than columns indicate +1 s.e.

brash is of importance for the soil effects, an interaction between brash treatment and fertiliser dose does not seem unlikely. In the Mangskog experiment, the amount of N in the needles in all the fertiliser treatments (0N-2400N) was fairly similar, namely 230 to 280 kg N $ha^{-1}$ (S. Jacobson, SkogForsk, personal communication, 2001). Thus, the potential leaching of N from the needles ought to have been similar between treatments, and no interaction between brash treatment and fertiliser dose was to be expected. Accordingly, a significant interaction between brash treatment and fertiliser dose was observed only for $Mg^{2+}$ in the soil solution. However, the interaction was complicated and not linearly related to the fertiliser dose. The amounts of dry matter and nutrients in the aboveground tree biomass, on the other hand, were negatively correlated to the fertiliser dose [21]. The analyses were complicated by the spatial variation in fertility within the Mangskog experiment[20]. The variation in fertility existed already when the experiment was established, and it ought to have primarily affected the interpretation of the effects of the main-plot treatments, i.e., the fertiliser dose.

The field-layer biomass increased rapidly as expected after clear felling. No significant difference in gross biomass production between the two brash treatments was found. Other studies on biomass production following brash removal have either reported increases or no significant effect on field-layer biomass[12,26]. The different results are most probably an effect of the prefelling conditions, as regards to overall nutrient status of the site. A number of studies have emphasised the importance of the field layer for retaining inorganic N after clear felling[19].

In the present study we did not separate the field-layer plants into species. A review reports that some species benefit from brash removal, e.g., *Vaccinium vitis-idaea* and *Calluna vulgaris*[27]. On the other hand, more nitrophilic species like *Chamaenerion angustifolium* and *Rubus ideaus* are reduced in cover by brash removal. Such a difference could be expected from an ecophysiological point of view. Slow-growing Ericaceuos plants that are unable to utilise $NO_3^-$ as an N source[28] cannot compete with fast-growing species in an $NO_3^-$ rich environment[29].

Measurements made in conjunction with felling revealed that the previous N fertilisations had caused a significant lowering of the C to N ratio with increasing fertiliser dose, mainly resulting from an increased retention of N in the humus layer[21]. Since N mineralisation and nitrification — at least to some extent — are related to the C to N ratio in the soil, it could be hypothesised that the amount of inorganic N in the soil should increase with increasing fertiliser dose. Such a pattern was found in a similar experiment situated on a much less fertile site in central Sweden (Billingsjön)[30]. No effect of previous N fertilisations could be detected at Mangskog, except for an increase in the $NO_3^-$ amount of the mineral soil at the highest fertiliser dose. This is in contrast with other findings where a large memory effect of previous N fertilisations has been reported[15,18]. However, the sites investigated in the other studies are much poorer than the Mangskog site, which indicates that the effect of previous N fertilisations on N availability after felling might be influenced by site fertility. Poor sites characterised by high C to N ratios might have a higher N-retention capacity than rich sites, resulting in a greater memory effect as regards to previous N fertilisation.

### ACKNOWLEDGMENTS

The present study was financed by the Swedish National Energy Administration and the Forestry Research Institute of Sweden. Important contributions to fieldwork were made by Hagos Lundström, Sten Nordlund, Mats Eriksson, Lars-Åke Dahl, and Carl-Olof Ingels. Gunnar Jansson gave valuable advice on the statistical analysis.

| | | |
|---|---|---|
| Received: | July | 17, 2001 |
| Revised: | August | 27, 2001 |
| Accepted: | August | 29, 2001 |
| Published: | October | 12, 2001 |
